# O10.1. DISORGANIZED GYRIFICATION NETWORK PROPERTIES DURING THE TRANSITION TO PSYCHOSIS

**DOI:** 10.1093/schbul/sby015.253

**Published:** 2018-04-01

**Authors:** André Schmidt, Tushar Das, Daniel Hauke, Fabienne Harrisberger, Lena Palaniyappan, Stefan Borgwardt

**Affiliations:** 1 University of Basel; 2 University of Western Ontario

## Abstract

**Background:**

There is urgent need to improve the limited prognostic accuracy of psychopathology-based classifications to predict the onset of psychosis in clinical high-risk (CHR) subjects for psychosis. However, as yet no reliable biological marker has been established to differentiate CHR subjects who will develop psychosis from those who will not. This study investigated abnormalities in graph-based gyrification connectome in CHR subjects and patients with first-episode psychosis (FEP) and tested the accuracy of this systems-based approach to predict the transition to psychosis among CHR individuals.

**Methods:**

44 healthy controls (HC), 63 at-risk mental state (ARMS) subjects without later transition to psychosis (ARMS-NT), 16 ARMS subjects with later transition (ARMS-T), and 38 antipsychotic-free patients with FEP were recruited from the specialized clinic for the early detection of psychosis at the Department of Psychiatry, University of Basel, Basel, Switzerland. Gyrification-based structural covariance networks (connectomes) were constructed to quantify global integration, segregation and small-worldness. Extremely randomized trees with repeated, nested cross-validation was performed to differentiate ARMS-T from ARMS-NT individuals. Permutation testing was used to assess the significance of classification performance measures.

**Results:**

Small-worldness is reduced in both ARMS-T and FEP patients, secondary to reduced integration and increased segregation in both groups. In addition, we also found that transitivity (segregation) was significantly higher in ARMS-T and FEP groups compared to both ARMS-NT and healthy controls. Using the connectome properties as features, we obtained a high classification accuracy of 90% (balanced accuracy: 81%, positive predictive value: 85%, negative predictive value: 92%.) All performance measures were highly significant as indicated by permutation tests (all p < 0.01).

**Discussion:**

Our findings suggest that there is poor integration in the coordinated development of cortical folding in patients who develop psychosis. This study further indicates that gyrification-based connectomes might be a promising means to generate systems-based measures from anatomical data that improves individual prediction of psychosis transition in CHR subjects.

